# Monocular Pedestrian 3D Localization for Social Distance Monitoring

**DOI:** 10.3390/s21175908

**Published:** 2021-09-02

**Authors:** Yiru Niu, Zhihua Xu, Ershuai Xu, Gongwei Li, Yuan Huo, Wenbin Sun

**Affiliations:** 1 College of Geoscience and Surveying Engineering, China University of Mining and Technology (Beijing), Beijing 100083, China; y.niu@student.cumtb.edu.cn (Y.N.); es.xu@student.cumtb.edu.cn (E.X.); ligw@aircas.ac.cn (G.L.); y.huo@student.cumtb.edu.cn (Y.H.); swb@cumtb.edu.cn (W.S.); 2 State Key Laboratory of Coal Resources and Safe Mining, China University of Mining and Technology (Beijing), Beijing 100083, China; 3 Key Laboratory of Quantitative Remote Sensing Information Technology, Aerospace Information Research Institute, Chinese Academy of Sciences, Beijing 100094, China

**Keywords:** three-dimensional localization, social distance monitoring, monocular camera, LiDAR point clouds

## Abstract

Social distancing protocols have been highly recommended by the World Health Organization (WHO) to curb the spread of COVID-19. However, one major challenge to enforcing social distancing in public areas is how to perceive people in three dimensions. This paper proposes an innovative pedestrian 3D localization method using monocular images combined with terrestrial point clouds. In the proposed approach, camera calibration is achieved based on the correspondences between 2D image points and 3D world points. The vertical coordinates of the ground plane where pedestrians stand are extracted from the point clouds. Then, using the assumption that the pedestrian is always perpendicular to the ground, the 3D coordinates of the pedestrian’s feet and head are calculated iteratively using collinear equations. This allows the three-dimensional localization and height determination of pedestrians using monocular cameras, which are widely distributed in many major cities. The performance of the proposed method was evaluated using two different datasets. Experimental results show that the pedestrian localization error of the proposed approach was less than one meter within tens of meters and performed better than other localization techniques. The proposed approach uses simple and efficient calculations, obtains accurate location, and can be used to implement social distancing rules. Moreover, since the proposed approach also generates accurate height values, exclusionary schemes to social distancing protocols, particularly the parent-child exemption, can be introduced in the framework.

## 1. Introduction

At the beginning of 2020, the COVID-19 pandemic swept the world, significantly impacting people’s lives and global economic development. While researchers have been developing therapeutics and vaccines for coronaviruses, social distancing is still recommended, at least until 2022, to minimize the risks of another outbreak [1]. Recent studies have shown that when more than one-meter distancing is maintained, the risk of transmission declines by about 82% [2]. In many countries, various rules and strategies have been introduced to observe and monitor interpersonal distancing in public places to curb the spread of the virus [3]. South Korea and India, for example, use Global Positioning System (GPS) data to monitor the movements of pedestrians to identify potential contacts. In addition, India has been utilizing smartphones to locate and monitor COVID-19 patients in target areas with the help of GPS and Bluetooth [4]. However, inside buildings, GPS is susceptible to signal attenuation and multipath effect, resulting in incorrect positioning and limiting its use in implementing social distancing.

The fundamental challenge in implementing social distancing and suppressing the spread of the virus is how to perceive people in 3D. Over the past decades, several emerging pedestrian 3D localization technologies have been introduced, from multi-sensor approaches to vision-based solutions. Guo et al. [5] put forward the Indoor Knowledge Graph Framework, integrating spatial information from various mobile phone sensors (including inertial sensor, Wi-Fi, and magnetic field) to achieve indoor positioning. Levchev et al. [6] used cameras, LiDAR, wireless sensors, inertial gyroscopes, and other sensors as data acquisition platforms and proposed a multi-sensor hybrid database configuration. Liang et al. [7] used different sensors to acquire image data and geographic location data simultaneously to construct an indoor 3D map with geographic coordinates. Zhang et al. [8] fused 3D point clouds with scene images to obtain the 3D positioning of outdoor scenes. However, as data acquisition platforms, most of these methods rely on a variety of sensors and have great dependence on the diversity of devices. In addition, some sensors (e.g., wireless) require multiple receiving antennas. Ultra-Wide Band (UWB) needs the target to wear tags or chips, which can be difficult to deploy in actual scenarios.

Stereovision is a relatively mature 3D localization technology, but our goal is to propose a solution that primarily uses monocular closed-circuit cameras. At present, monocular vision research mainly focuses on the 3D position estimation of vehicles [9,10] since pedestrians have varying heights and body shapes and lack enough attribute information. Most traditional methods for pedestrian 3D localization using monocular cameras rely on known information about the scene or pedestrian to obtain the unknown metrics. For example, Gunel et al. [11] and Das and Meher [12] used medical statistical data to locate pedestrians based on the relationship of human height and body parts (or stride length). Using physical constraints, Bieler et al. [13] converted pixel measurements into human height through a simple motion trajectory analysis (jumping or running). In most cases, this estimation approach indirectly obtains the pedestrian’s 3D position using some other metrics, which may result in error propagation.

With developments in artificial intelligence in recent years, various studies have used machine learning technology for monocular pedestrian 3D localization. For example, Deng et al. [14] proposed an end-to-end trainable neural network, which only uses synthetic data to obtain stereo images and body pose estimation. Bertoni et al. [15] used a feedforward neural network to predict confidence intervals of 2D human posture and 3D position based on the loss function of Laplace distribution. Bertoni et al. [16] also used a neural network to predict confidence intervals and estimate the 3D position and orientation of pedestrian. However, in most of these studies, pedestrians are assumed to be of equal height, resulting in a task error of about one meter for pedestrians 20 m away.

Visual-based technology has allowed the remote determination of pedestrians’ posture and acquisition of texture information. However, due to the limitations of having only one projection line corresponding to each object point and the lack of depth value, additional information is needed to convert 2D images into 3D. In this context, we propose an effective alternative to achieve three-dimensional positioning. We used terrestrial LiDAR to capture 3D maps to estimate the parameters of the monocular camera. Since the pedestrian is always perpendicular to the ground, we could determine the 3D localization of pedestrians. Our method focuses on solving the limitations of plane positioning and improving the applications of traditional photogrammetry in three-dimensional space.

## 2. Proposed Method

**Figure 1:**
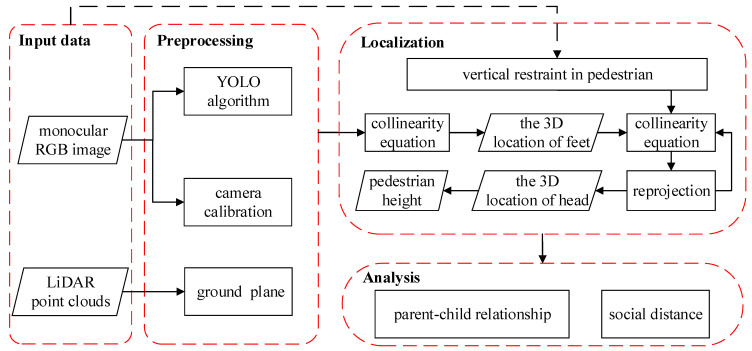
The flowchart of the proposed method.

A monocular pedestrian 3D localization for social distance monitoring framework is proposed in this study. Figure 1 presents the general flowchart of the proposed method. The proposed 3D localization approach includes three main parts: monocular camera calibration (preprocessing), pedestrian 3D localization (localization), and social distance calculation (analysis).

### 2.1. Preprocessing

#### 2.1.1. Camera Calibration

In computer vision, camera calibration is used to determine the camera position and establish the imaging model. Then the relative relationship between the space coordinate system and the image coordinate system can be obtained. To obtain 3D positioning based on monocular images, camera calibration is needed to recover internal (i.e., the focal length f and the pixel coordinates of the principal point ($u_{0},\;v_{0}$)) and external parameters (i.e., translation vector T and rotation matrix R). For every scenario, at least six pairs of feature points are manually selected from the terrestrial point clouds and monocular image to obtain the 2D-3D matching relationship. The 2D and 3D point coordinates are respectively denoted by ($u_{i},\;v_{i}$) and ($X_{i}$,$\;Y_{i},\;Z_{i}$) where i represents different matching points. We use direct linear transformation (DLT) [17] to acquire the camera orientation. The parameters can be formulated as:

(1) $$\left[\begin{array}{c} u_{i}\\ v_{i}\\ 1 \end{array}\right]=\left[\begin{array}{cccc} f & 0 & u_{0} & 0\\ 0 & f & v_{0} & 0\\ 0 & 0 & 1 & 0 \end{array}\right]\left[\begin{array}{cc} R & T\\ 0^{T} & 1 \end{array}\right]\left[\begin{array}{c} X_{i}\\ Y_{i}\\ \begin{array}{c} Z_{i}\\ 1 \end{array} \end{array}\right]$$

Express it as an equation for the unknown quantity $\;l_{j}\left(j=1,2,\ldots 12\right)$:

(2) $$\left[\begin{array}{c} u_{i}\\ v_{i}\\ 1 \end{array}\right]=L\left[\begin{array}{c} X_{i}\\ Y_{i}\\ \begin{array}{c} Z_{i}\\ 1 \end{array} \end{array}\right]=\left[\begin{array}{cccc} l_{1} & l_{2} & l_{3} & l_{4}\\ l_{5} & l_{6} & l_{7} & l_{8}\\ l_{9} & l_{10} & l_{11} & l_{12} \end{array}\right]\left[\begin{array}{c} X_{i}\\ Y_{i}\\ \begin{array}{c} Z_{i}\\ 1 \end{array} \end{array}\right]$$

The homogeneous coordinate $l_{12}\;$ does not change the values. We set $\;l_{12}=1$. Then substitute the i (i≥6) pairs of matching points into the Equation (2):

(3) $$\left[\begin{array}{ccccccccccc} X_{1} & Y_{1} & Z_{1} & 1 & 0 & 0 & 0 & 0 & u_{1}X_{1} & u_{1}Y_{1} & u_{1}Z_{1}\\ 0 & 0 & 0 & 0 & X_{1} & Y_{1} & Z_{1} & 1 & v_{1}X_{1} & v_{1}Y_{1} & v_{1}Z_{1}\\ X_{2} & Y_{2} & Z_{2} & 1 & 0 & 0 & 0 & 0 & u_{2}X_{2} & u_{2}Y_{2} & u_{2}Z_{2}\\ 0 & 0 & 0 & 0 & X_{2} & Y_{2} & Z_{2} & 1 & v_{2}X_{2} & v_{2}Y_{2} & v_{2}Z_{2}\\ \vdots & \vdots & \vdots & \vdots & \vdots & \vdots & \vdots & \vdots & \vdots & \vdots & \vdots \\ X_{i} & Y_{i} & Z_{i} & 1 & 0 & 0 & 0 & 0 & u_{i}X_{i} & u_{i}Y_{i} & u_{i}Z_{i}\\ 0 & 0 & 0 & 0 & X_{i} & Y_{i} & Z_{i} & 1 & v_{i}X_{i} & v_{i}Y_{i} & v_{i}Z_{i} \end{array}\right]\left[\begin{array}{c} l_{1}\\ l_{2}\\ \begin{array}{c} l_{3}\\ l_{4}\\ \begin{array}{c} l_{5}\\ l_{6}\\ \begin{array}{c} l_{7}\\ l_{8}\\ \begin{array}{c} l_{9}\\ \begin{array}{c} l_{10}\\ l_{11} \end{array} \end{array} \end{array} \end{array} \end{array} \end{array}\right]=\left[\begin{array}{c} -u_{1}\\ -v_{1}\\ \begin{array}{c} -u_{2}\\ -v_{2}\\ \begin{array}{c} \vdots \\ -u_{i}\\ -v_{i} \end{array} \end{array} \end{array}\right]$$

Then write Equation (3) as:

(4) AL=b

Solve for L using the following formula:

(5) $$L={\left(A^{T}A\right)}^{-1}A^{T}b$$

We can then use mathematical operations to get the values of the internal and external parameters.

#### 2.1.2. Detector

According to Section 2.1.1, it is very important to obtain accurate 2D pixel coordinates of the pedestrian. The YOLO object detector [18] is used for pedestrian detection in monocular image coordinates. Only one CNN operation is needed to realize the end-to-end prediction. The algorithm uses the whole monocular image as input to the network and simultaneously predicts the target area and its category. In this paper, only the detection results of people are selected. This operation would provide the midpoint pixel coordinates of the detection box’s upper and lower edges, which would then be used to represent the head and feet of the pedestrian.

#### 2.1.3. Extraction of the Vertical Coordinates of the Ground

The gyroscope in the LiDAR helps maintain the *z*-axis of the point cloud coordinate system in a vertical orientation, and a pedestrian remains upright while walking. The 3D coordinates of pedestrian feet are calculated with this constraint condition. We crop the terrestrial point clouds based on the view range of the monocular image. Then a grid-based point cloud segmentation algorithm is used to obtain the ground plane. The algorithm first projects the 3D point clouds onto the grid map, with a single grid regarded as the minimum computing unit. Then, the statistical characteristics of the elevation direction in each grid unit, including extremum, mean, and variance are computed. The average height difference of a grid is compared with its adjacent grids. Grids with small height differences are marked as potential ground location. Adjacent potential ground locations constitute a connected domain. The connected domain with the largest area can be regarded as the ground. Then from the point clouds, we can acquire the vertical coordinates of the ground, which provides necessary data for the subsequent pedestrian 3D localization.

### 2.2. 3D Localization

The midpoint *t* of the pedestrian bounding box’s upper edge (i.e., the position of the pedestrian’s head) and the midpoint *b* of its lower edge (i.e., the position of pedestrian’s feet) are used as marking points. For pedestrians who only appear in a single frame of the monocular camera, their 3D coordinates cannot be directly obtained from a single uncalibrated image and would have to be solved using additional information. Given the presence of a gyroscope in LiDAR, the *z*-axis in its coordinate system would always be perpendicular to the ground. Therefore, $Z_{g}$ is introduced to represent the *z*-axis coordinate of the ground plane in the point cloud coordinate system. Based on the camera calibration and posture recovery, the 3D coordinates $\;\left(X_{b},Y_{b},Z_{b}\right)$ of the pedestrian feet point *b* can be obtained using the space intersection:

(6) $$\{\begin{array}{c} X_{b}=\frac{\left(Z_{g}-Z_{S}\right)\left(a_{1}*\frac{u_{0}-u_{b}}{f}+a_{2}*\frac{v_{b}-v_{0}}{f}+a_{3}\right)}{c_{1}*\frac{u_{0}-u_{b}}{f}+c_{2}*\frac{v_{b}-v_{0}}{f}+c_{3}}+X_{S}\\ Y_{b}=\frac{\left(Z_{g}-Z_{S}\right)\left(b_{1}*\frac{u_{0}-u_{b}}{f}+b_{2}*\frac{v_{b}-v_{0}}{f}+b_{3}\right)}{c_{1}*\frac{u_{0}-u_{b}}{f}+c_{2}*\frac{v_{b}-v_{0}}{f}+c_{3}}+Y_{S}\\ Z_{b}=Z_{g} \end{array}$$

where ($u_{b}$,$v_{b}$) are the pixel coordinates of foot-point *b*, $X_{S}$, $Y_{S}$, and $Z_{S}$ are the line elements in the translation vector T, and $a_{i}$, $b_{i}$, and $c_{i}$ (i = 1, 2, 3) are the elements in rotation matrix R. The reprojection error $\left(\Delta u_{t},\Delta v_{t}\right)\;$ of point *t* can then be calculated based on the inherent condition that the pedestrian is always perpendicular to the ground. The pixel coordinates are computed using the expressions:

(7) $$\sqrt{{\left(X_{b}-X_{t}\right)}^{2}+{\left(Y_{b}-Y_{t}\right)}^{2}}=0$$

(8) $$\left[\begin{array}{c} \Delta u_{t}\\ \Delta v_{t} \end{array}\right]=\left[\begin{array}{c} u_{t}\\ v_{t} \end{array}\right]-\left[\begin{array}{c} u_{t}^{0}\\ v_{t}^{0} \end{array}\right]=\left[\begin{array}{c} \frac{f\left(a_{1}X_{t}+b_{1}Y_{t}+c_{1}Z_{t}+X_{S}\right)}{a_{3}X_{t}+b_{3}Y_{t}+c_{3}Z_{t}+Z_{S}}\\ \frac{f\left(a_{2}X_{t}+b_{2}Y_{t}+c_{2}Z_{t}+Y_{S}\right)}{a_{3}X_{t}+b_{3}Y_{t}+c_{3}Z_{t}+Z_{S}} \end{array}\right]+\left[\begin{array}{c} u_{0}\\ v_{0} \end{array}\right]-\left[\begin{array}{c} u_{t}^{0}\\ v_{t}^{0} \end{array}\right]$$

where ($u_{t}$, $v_{t}$) are the pixel coordinates of the head point *t* by central projection, and ($u_{t}^{0}$,$\;v_{t}^{0}$) are the pixel coordinates of point *t* that were directly detected. Based on Taylor’s theorem, if P=[X, Y, Z], then

(9) $$F\left(P\right)\approx F\left(P_{0}\right)+\nabla F{\left(P_{0}\right)}^{T}\left(P-P_{0}\right)$$

Accordingly, the reprojection error in Equation (8) is sorted into an equation of $Z_{t}$ given by the formula:

(10) $$\nabla F\left(P\right)=\frac{\partial F}{\partial P}=\left[\begin{array}{cc} \frac{\partial u_{t}}{\partial Z_{t}} & \frac{\partial v_{t}}{\partial Z_{t}} \end{array}\right]$$

(11) $$\left[\begin{array}{c} \frac{\partial \Delta u_{t}}{\partial Z_{t}}\\ \frac{\partial \Delta v_{t}}{\partial Z_{t}} \end{array}\right]\Delta Z_{t}+\left[\begin{array}{c} \Delta u_{t}\\ \Delta v_{t} \end{array}\right]=0$$

In Equation (11), matrix operation is performed to calculate the correction $\Delta Z_{t}$. After several iterations, the head 3D coordinates ($X_{t}\;,Y_{t}\;,Z_{t}$) satisfying the precision requirements can then be obtained. After the 3D coordinates of the pedestrian’s head and feet are obtained, the height *h* can be calculated using the formula:

(12) $$h=\sqrt{{\left(X_{b}-X_{t}\right)}^{2}+{\left(Y_{b}-Y_{t}\right)}^{2}+{\left(Z_{b}-Z_{t}\right)}^{2}}$$

### 2.3. Social Distance Monitoring

The midpoint *b* at the bottom edge of the pedestrian bounding box is used as the reference point (i.e., feet location). After calculating the 3D position, the interpersonal distance in a homogeneous space is determined using the person-to-person Euclidean distance [3]. If the interpersonal distance is more than the threshold value *r* (here, *r* is set to one meter based on the epidemic prevention and control requirements), the social distancing requirement is satisfied.

(13) $$\left(b_{i},b_{j},r\right)=\{\begin{array}{c} 0\;\;\;\mathrm{if}\sqrt{{\left(X_{i}-X_{j}\right)}^{2}+{\left(Y_{i}-Y_{j}\right)}^{2}+{\left(Z_{i}-Z_{j}\right)}^{2}}\leq r\\ 1\;\;\;\mathrm{if}\sqrt{{\left(X_{i}-X_{j}\right)}^{2}+{\left(Y_{i}-Y_{j}\right)}^{2}+{\left(Z_{i}-Z_{j}\right)}^{2}}>r \end{array}$$

### 2.4. Performance Evaluation

For quantitative evaluation, we used two indicators: average localization error (ALE) and average localization precision (ALP). ALE is the average Euclidean distance between the predicted positions of feature points and their real positions. Here, we used the pedestrians’ heads and feet as feature points for reference. ALE reflects the stability and accuracy of the proposed method and is given by the expression:

(14) $$\;\mathrm{ALE}=\frac{1}{n}\sum_{k=1}^{n}\sqrt{{\left({\hat{X}}_{k}-X_{k}\right)}^{2}+{\left({\hat{Y}}_{k}-Y_{k}\right)}^{2}+{\left({\hat{Z}}_{k}-Z_{k}\right)}^{2}}$$

where *n* is the number of feature points, ($X_{k},Y_{k},Z_{k}$) are the predicted positions of feature points, and (${\hat{X}}_{k}$,$\;{\hat{Y}}_{k}$, ${\hat{Z}}_{k}$) are their real positions.

The ALP indicator, defined by Xiang et al. [19], was originally used in evaluating car classification. In order to ensure the detection effect of YOLO, images with relatively large number of pedestrians and less occlusion between pedestrians were selected from the dataset. Then, the localization errors of all pedestrians detected in the selected images are calculated. The probabilities of errors less than the threshold *r* were used as the ${\mathrm{ALP}}_{r}$ values in our proposed method. ${\mathrm{ALP}}_{r}$ can be calculated using the equation:

(15) $${\mathrm{ALP}}_{r}=\frac{1}{n}\sum_{k=1}^{n}P\left(k,r\right)\;\;\;\;\;r\in \left\{0.5,\;1,\;2\right\}$$

where P(k,r) is the localization precision. The localization prediction is considered correct if the error between the predicted distance and the ground truth is within the given threshold.

## 3. Experiments and Results

For the experiments, we used two test data (i.e., CUMTB-Campus and KITTI) to evaluate the performance of our proposed method. First, the CUMTB-Campus dataset was used to show the positioning accuracy in different conditions. Second, the KITTI dataset was used to compare and contrast our proposed method against other localization methods.

### 3.1. Datasets

#### 3.1.1. Self-Collected Data in CUMTB

**Figure 2:**
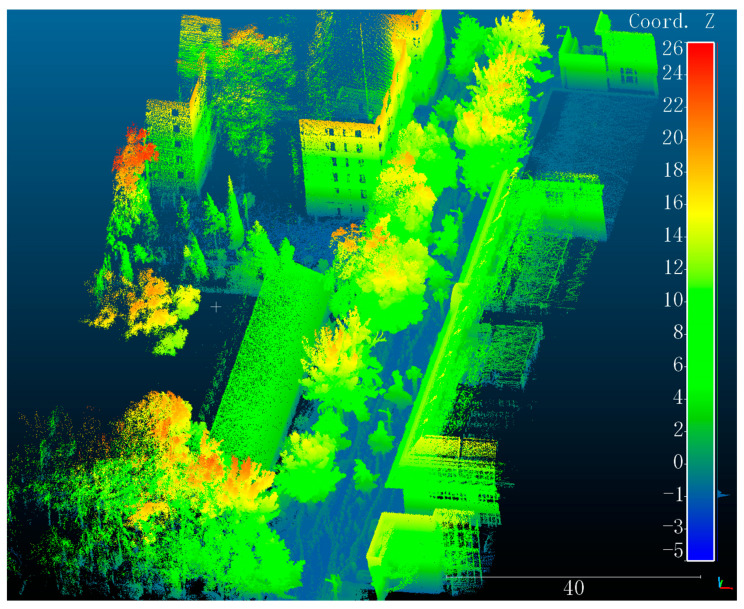
LiDAR point clouds colored based on height in CUMTB-Campus.

Along the main road of CUMTB, we used a hand-held LiDAR scanner GeoSLAM-ZEB-HORIZON to collect 3D point clouds of the campus. In standard conditions, the measuring distance of this scanner is 100 m, and the relative accuracy is 1.5–3 cm. Figure 2 gives an example of our self-collected point clouds, which colored based on height. Video images were obtained simultaneously. The data includes major buildings such as teaching buildings, libraries, ethnic buildings, complex buildings, roads, and other facilities, covering about 80,000 square meters of the campus. At the same time, the video images of surveillance cameras widely distributed in the campus were obtained. In addition, non-metric camera was also used to take photos of pedestrians in some indoor and outdoor scenes on campus (such as teaching buildings, outside the library). Based on the above data, we built our self-collected dataset CUMTB-Campus. Specific parameters in standard conditions are shown in Table 1.

#### 3.1.2. The KITTI Dataset

As the largest dataset for evaluating computer vision algorithms in autonomous driving scenarios, KITTI [20] contains real image data from urban, rural, and highway scenes, with up to 15 cars and 30 pedestrians per image. The dataset also has varying degrees of occlusion and truncation. In this paper, several unobstructed images of pedestrians and corresponding point clouds were selected for testing. Table 1 summarizes the device parameters of the datasets used in the study.

### 3.2. Implementation Details and Results

#### 3.2.1. Implementation Details and Results for the CUMTB-Campus Dataset

The CUMTB-Campus experiment was designed based on the process described in Section 2. To calibrate the camera, non-coplanar feature points were selected from the image and point clouds, respectively. Using these feature points, the camera elements on interior and exterior orientation were obtained. Here we select the data of two different scenarios to show our positioning results.

**Figure 3:**
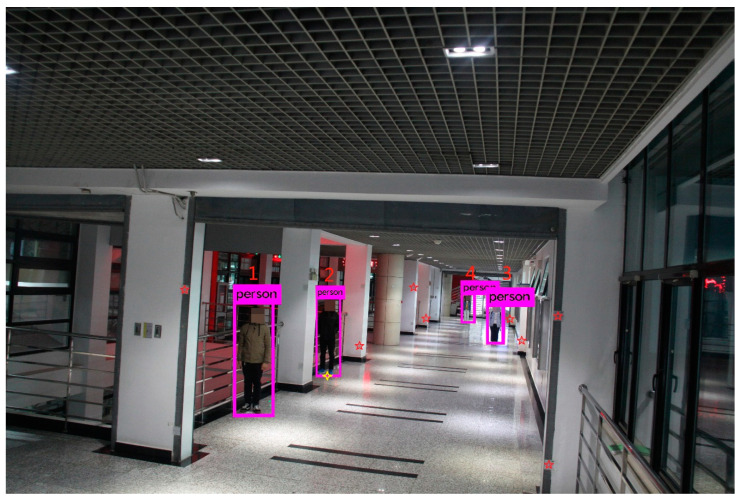
Scene 1 in CUMTB-Campus.

1. Scene 1:

Scene 1 was an indoor scenario in the teaching building of CUMTB. The image with 15.1 megapixel resolution was taken by Canon EOS 50D on 24 October 2019, while the GeoSLAM-ZEB-HORIZON scanner acquired LiDAR point clouds along the corridor in the same region. We extracted the coordinates for the vertical direction of the ground plane where the pedestrian is situated from the point clouds. Then, as shown in Figure 3, the YOLO algorithm was used to obtain the pixel coordinates for each pedestrian detection box.

Camera calibration is realized based on Section 2.1.1. As shown in Figure 3, in order to facilitate the selection of feature points, we stick a number of reflectors in the scene. Here, the center points of eight uniformly distributed reflectors are manually selected from the point clouds and monocular image as matching points (represented by red five-pointed stars). The 2D pixel coordinates and 3D coordinates of matching points are read and substituted into Equation (3) to calculate the internal and external parameters of the monocular image. The results are as follows: f = 3938.104 pixels, the pixel coordinates of the principal point ($u_{0},\;v_{0}$) are (2386.167, 1596.880), the translation vector T=(3.563,5.210,2.058) meters, the rotation matrix $R=\left[\begin{array}{ccc} 0.167 & -0.985 & 0.050\\ 0.058 & 0.041 & 0.997\\ -0.984 & -0.170 & -0.050 \end{array}\right]$. Based on the obtained parameters, test points are selected to verify the calibration accuracy. Taking the yellow four-pointed star as an example, its reprojected pixel coordinates are (2064.925, 2392.275) and the reprojection errors are 3.079 pixels.

Then using our proposed approach, each pedestrian’s 3D position and height can be obtained using a single RGB image. Table 2 shows pedestrians’ localization and height errors with different distances from the camera in Scene 1 of the CUMTB-Campus. The social distance errors between adjacent pedestrians are also listed.

**Figure 4:**
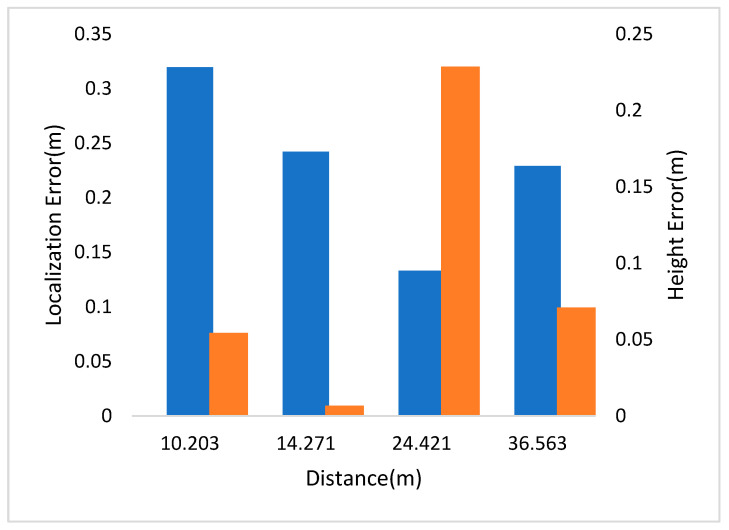
Localization and height error of Scene 1 in CUMTB-Campus.

In Figure 4, the left and right vertical axes represent the localization and height errors of four pedestrians in Scene 1 at different distances to the camera. The experimental results show that the localization error was less than half a meter within the 36-m vision range.

**Figure 5:**
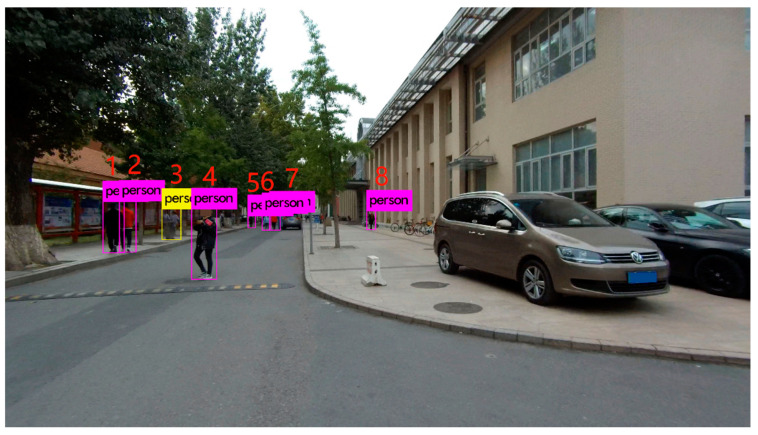
Scene 2 in CUMTB-Campus.

2. Scene 2:

Scene 2 was an outdoor scene on the main road besides the library of the CUMTB. The image with 8.3 megapixel resolution was taken by GeoSLAM-ZEB-HORIZON scanner on 22 October 2019. The scanner also acquired LiDAR point clouds along the same road. There are more people in this scene, and they are in different positions including people overlapping. There are also several pedestrians on the steps. As shown in Figure 5, the YOLO algorithm was used to obtain the pixel coordinates for each pedestrian detection box.

As can be seen from Figure 5, there are two pedestrians walking side by side and partially covered, and our detection algorithm only obtains one detection box (the yellow one). In order to evaluate the positioning accuracy rather than the 2D detection accuracy, the detection box is treated as a pedestrian for 3D localization. Then, using the above method, we can obtain the 3D position and height of pedestrians. Table 3 shows the localization and height errors of eight pedestrians in Scene 2. Similarly, we also list the social distance errors between adjacent pedestrians.

**Figure 6:**
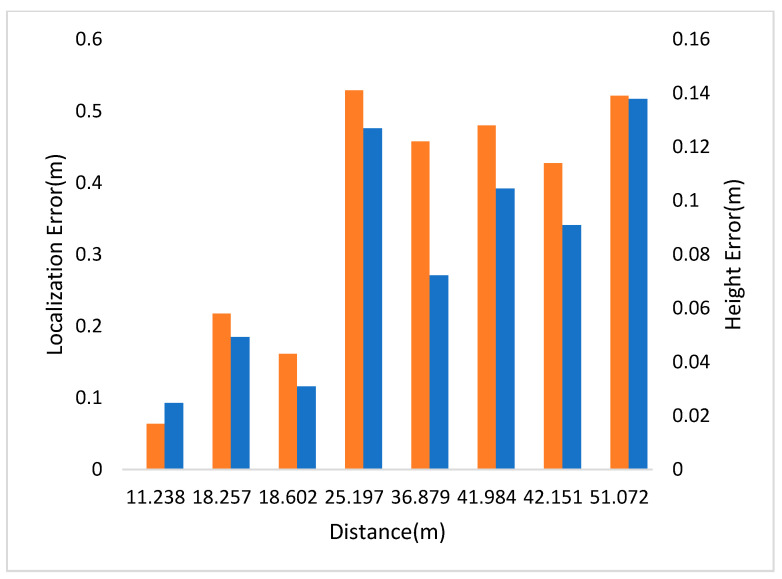
Localization and height error of Scene 2 in CUMTB-Campus.

In Figure 6, the left and right vertical axes represent the localization and height errors of eight pedestrians in Scene 2 at different positions. In this scenario, pedestrian 5 has the largest localization error because he is more than 50 m away from the camera. The second one is the yellow box due to the occlusion. The maximum relative error of social distance is 0.207 m.

#### 3.2.2. Implementation Details and Results for the KITTI Dataset

**Figure 7:**
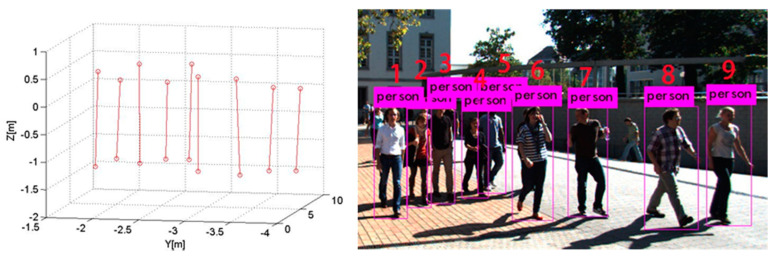
Scene 3 and pedestrian localization results.

From the KITTI dataset, we randomly selected multiple images of fully visible pedestrians. Using the camera calibration parameters provided by the dataset, the three-dimensional coordinates and height of pedestrians were obtained. Figure 7 shows Scene 3 selected from the KITTI dataset and nine pedestrian localization results. Table 4 summarizes the localization errors of the pedestrians’ feet for Scene 3 and their height and social distancing errors.

**Figure 8:**
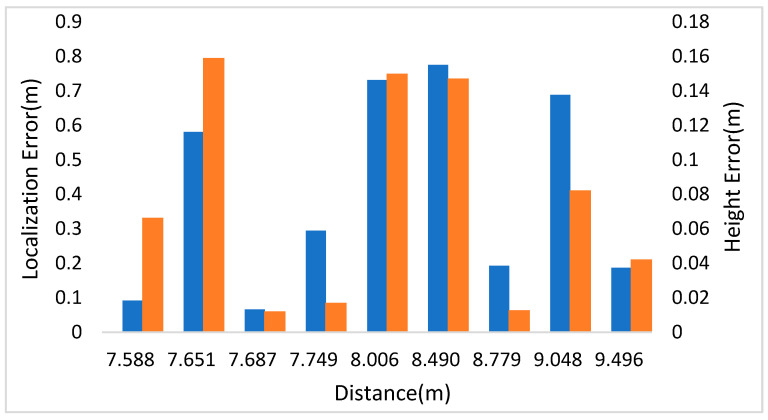
Localization and height error of Scene 3 in KITTI.

In Figure 8, the left and right vertical axes represent the localization and height errors of nine pedestrians in Scene 3. Figure 8 shows that the biggest localization and height errors of Scene 3 were 0.775 m and 0.159 m. Among them, pedestrian 2 and pedestrian 4 are affected by other pedestrians, leading to a large error in two-dimensional detection results, affecting the positioning accuracy. The maximum relative error of social distance is 0.246 m.

### 3.3. Methods Comparison

We then compared our positioning results with three monocular 3D localization methods based on the KITTI dataset, i.e., Mono3D [21], MonoDepth [22], and Monoloco [15]. We also evaluated our results with a binocular approach, i.e., 3DOP [23]. Its input is a stereo image pair, which uses 3D information and a context model specific to the autonomous driving field to obtain a 3D bounding box. As shown in Table 5, we evaluate our method with other localization results in [15]. Due to limitations of our implementation method, five images (including Scene 3) were randomly selected from the KITTI dataset containing 25 fully visible pedestrians at different distances. The 3D coordinates of 50 pedestrian points (each pedestrian contains two points, i.e., head and feet) were then calculated, and the accuracy was evaluated using the ${\mathrm{ALP}}_{r}$(*r* = 0.5, 1, 2) and ALE mentioned above.

As shown in Table 5, the average localization error of our proposed method in the test data was within one meter, i.e., 0.20 m. Among them, 78% of the localization errors were within 0.5 m. With the aid of high-precision point clouds, our proposed approach outperformed other monocular localization methods, such as the Monoloco [15]. This method uses detected 2D joints of a network as input and generates the 3D location with confidence interval. However, this method assumes that all pedestrains have the same height, resulting in error-prone estimates. Then, our proposed method achieved comparable performance with the 3DOP, which uses stereo images for training and testing. However, our method does not require stereo images to obtain better accuracy and does not require datasets for training. In addition, our proposed method was able to calculate pedestrians’ height. In the selected images, the average height error of twenty-five pedestrians was 0.05 m.

### 3.4. Social Distance Monitoring

Based on COVID-19 prevention and control protocols, one-meter social distancing should be observed between pedestrians. However, this rule on social distancing is not considered absolute. For instance, if adults and children walk side by side, the rule on social distancing is bypassed even if the distance between them is less than one meter. To account for this special circumstance, an exclusion scheme for parent and child pedestrians, determined based on height, is adopted. We adopt the standard of free admission for children in most public places (such as tourist attractions, amusement parks, cinemas, etc.) in China and choose 1.2 m as the reference height for children. At the same time, according to the relevant statistical data [25], the average height of 1.715 m was selected as the adult height reference. So in the proposed scheme, pedestrians walking side by side with a height difference of more than 51.5 cm would be considered parent and child.

**Figure 9:**
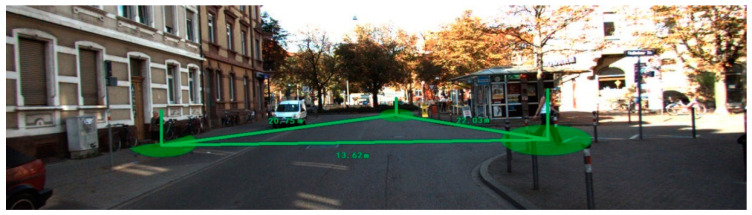
An example scene showing acceptable social distancing.

**Figure 10:**
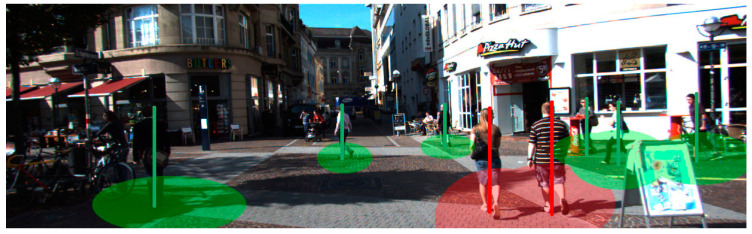
An example scene with various social distancing types.

Since our proposed localization method includes 3D location determination and height estimation, the parent-and-child exemption could be executed. In Figure 9 and Figure 10, the 3D position and height of pedestrians are marked in different colors. Red is used to mark violations to social distancing rules and green pertains to adherence to social distancing protocols.

## 4. Discussion

This study investigated the monocular pedestrian 3D localization for social distance monitoring. According to the experimental results in Section 3, the pedestrian localization and height errors obtained by the proposed method are mainly affected by the following factors:

(1). 1. The quality of point clouds:

Mobile mapping systems (MMS) devices are used to acquire the point clouds in both tests (CUMTB-Campus and KITTI). When there are pedestrians or other moving objects in the scenes, the integrity of point clouds may be affected. In addition, the observation accuracy of LiDAR scanner is also related to the scanning distance and the fine degree of scanning. In the process of camera calibration, 3D coordinates of feature points are obtained from point clouds. In addition, point cloud segmentation is needed to obtain the vertical coordinate of the ground plane. Therefore, point cloud accuracy will have a certain impact on camera calibration and localization accuracy. However, in the proposed method, this factor has little influence on the final result.

(2). Select the corresponding point pairs:

As described in Section 2.1.1, at least six pairs of matching points should be selected from the point clouds and monocular image for DLT to obtain the camera parameters. As shown in Figure 3, eight evenly distributed feature points are selected around pedestrians. Then, the number and location distribution of the selected points will have a certain impact on the calibration results.

(3). Distance from pedestrian to the projection center of monocular image:

In the indoor scene shown in Figure 3, the distance differences between the pedestrians and the camera are not too large. The final result is mainly affected by other factors such as calibration accuracy and research environment. Thus, the localization and height errors are not linearly connected to the distances amplitude. As shown in Figure 5, some pedestrians are far from the camera. The YOLO algorithm may fail to obtain the pedestrian detection box or the coordinates of the detection box are inaccurate. In addition, the actual distance corresponding to a pixel is larger. In this case, the accuracy is mainly affected by distance, and the localization and height errors are approximately linearly related to distance.

(4). Environmental situation:

Our method may be influenced by the environmental situation, such as whether there are steps or slopes in the scene and whether pedestrians are blocked or truncated. As shown in Figure 5, it can be seen that for pedestrians in different height ground planes, the proposed method can still achieve pedestrian 3D localization. However, since our approach requires the assistance of terrestrial point clouds, it is difficult to extract the ground plane of pedestrians if there are slopes or steps with large inclination in the scene. In addition, by analyzing the experimental results, it can be found that when pedestrians are blocked or truncated, the YOLO algorithm may fail to obtain accurate detection box coordinates. At this time, the localization error mainly comes from 2D detection. The use of additional auxiliary information may help solve this problem. For example, Gunel et al. [11] confirmed that human height could be inferred from images by obtaining joint posture, bone length ratio, and facial features. Kundegorski and Breckon [26] used infrared images for pedestrian tracking and obtained 3D locations based on the medical statistics assuming adult height.

To sum up, the accuracy of our proposed method relies on some external factors. During data collection, the quality of point clouds should be ensured as far as possible, and a certain number of uniformly distributed matching points can improve the localization accuracy. In general, our method is a simple and accurate monocular pedestrian 3D localization and social distance monitoring solution. The proposed approach can be extended and applied to other fields, such as automatic piloting, crowd analysis, fire safety, and merchant passenger flow statistics.

## 5. Conclusions

An innovative and accurate approach using monocular RGB images to obtain pedestrian localization and assess social distancing is proposed in this paper. The proposed method takes advantage of the inherent condition that the pedestrian is always perpendicular to the ground plane. Our method improves on the current localization in various aspects. First, our method only needs to obtain the pedestrian detection box without extracting the skeleton. Second, it does not rely on the base station, network or tag constraints, and can obtain the pedestrian’s 3D position based on monocular image and terrestrial point clouds, and the precision meets the social distancing protocol of one meter. Finally, since the proposed localization approach also generates accurate height values, exclusionary schemes to social distancing protocols, particularly the parent-child exemption, can be introduced in the framework. Using height difference between pedestrians, the proposed approach can identify violations of social distancing rules and determine cases most likely covered by the parent-child exemption. During the epidemic prevention and control period, for some places requiring key prevention and control, such as schools, hospitals, shopping malls, LiDAR scanners can be used to obtain their 3D point clouds. During data collection, only the point clouds on the ground containing the pedestrian accessible area need to be scanned. Then, using widely distributed surveillance cameras, 3D positioning of pedestrians in the range of view can be obtained. In future works, we will continue to study the pedestrian 3D localization when they are blocked or truncated and try to solve this problem by obtaining the pedestrian body proportions through skeleton detection. While social distancing is important to minimize community transmission of the virus, a moderate amount of social interaction is also necessary. Using statistical analysis of the spatial-temporal data of pedestrians, high-risk areas for virus transmission and community spread can be identified. This would help authorities reevaluate pedestrian traffic design and implement more proactive measures to mitigate risks.

## Figures and Tables

**Table 1:** Equipment parameters of two datasets.

Equipment Type	CUMTB-Campus		KITTI	
	Canon EOS 50D	GeoSLAM-ZEB-HORIZON	FL2-14S3C-C	Velodyne 64-Wire 3D Laser Scanner
Mode	Image	Point clouds	Image	Point clouds
Resolution	4752 × 3168	1.5–3 cm	1224 × 370	2 cm

**Table 2:** Pedestrian localization and height errors of Scene 1 in CUMTB-Campus.

Pedestrian Number	Distance (m)	Localization Error (m)	Height Error (m)	Social Distance			
				Adjacent Pedestrian	Truth Value (m)	Calculated Value (m)	Error (m)
1	10.203	0.320	0.054	—	—	—	—
2	14.271	0.242	0.007	1~2	4.832	4.770	−0.062
3	24.421	0.133	0.229	2~3	10.266	10.199	−0.067
4	36.563	0.229	0.071	3~4	13.122	13.209	0.087

**Table 3:** Pedestrian localization and height errors of Scene 2 in CUMTB-Campus.

Pedestrian Number	Distance (m)	Localization Error (m)	Height Error (m)	Social Distance			
				Adjacent Pedestrian	Truth Value (m)	Calculated Value (m)	Error (m)
1	18.257	0.185	0.058	—	—	—	—
2	18.602	0.116	0.043	1~2	0.682	0.589	−0.093
3	25.197	0.476	0.141	2~3	8.059	8.176	0.117
4	11.238	0.093	0.017	3~4	14.709	14.548	−0.161
5	51.072	0.517	0.139	4~5	41.132	40.925	−0.207
6	42.151	0.341	0.114	5~6	9.171	9.367	0.196
7	41.984	0.392	0.128	6~7	0.676	0.558	−0.118
8	36.879	0.271	0.122	7~8	6.205	6.112	−0.093

**Table 4:** Pedestrian localization and height errors of Scene 3 in KITTI.

Pedestrian Number	Distance (m)	Localization Error (m)	Height Error (m)	Social Distance			
				Adjacent Pedestrian	Truth Value (m)	Calculated Value (m)	Error (m)
1	7.749	0.295	0.017	—	—	—	—
2	8.490	0.775	0.147	1~2	0.868	0.688	−0.180
3	8.779	0.193	0.012	2~3	0.632	0.860	0.228
4	9.048	0.688	0.082	3~4	0.683	0.585	−0.098
5	9.496	0.187	0.042	4~5	0.606	0.852	0.246
6	7.588	0.092	0.066	5~6	2.110	2.007	−0.103
7	7.687	0.066	0.012	6~7	0.929	0.930	0.001
8	7.651	0.581	0.159	7~8	1.102	1.216	0.114
9	8.006	0.731	0.150	8~9	0.677	0.643	−0.034

**Table 5:** Comparing our proposed method against baseline results on the KITTI dataset.

Method	Type	$ALP_{r}\;(\%)$			ALE (m)
		*r* = 0.5 m	*r* = 1 m	*r* = 2 m	Fully Visible
Mono3D	Mono	13.2	23.2	38.9	2.32
MonoDepth + PifPaf [24]	Mono	20.5	35.3	50.6	1.69
Monoloco-trained on KITTI	Mono	29.0	49.6	71.2	0.98
3DOP	Stereo	41.4	54.9	63.2	0.71
Our proposed method	Mono	78.0	100.0	100.0	0.20

## Data Availability

Open-source dataset KITTI is available from Karlsruhe Institute of Technology at http://www.cvlibs.net/datasets/kitti/raw_data.php accessed on 25 May 2021. Self-collected dataset CUMTB-Campus that support the findings of this study are available from the corresponding author upon reasonable request.
